# Lepromatous Leprosy and Charcot Neuroarthropathy of Insensate Feet: A Case Report

**DOI:** 10.7759/cureus.61362

**Published:** 2024-05-30

**Authors:** Gavin T Kress, Mark Swerdlow, Laura Shin

**Affiliations:** 1 Vascular Surgery, Keck School of Medicine of the University of Southern California, Los Angeles, USA

**Keywords:** mycobacterium leprae, osteolytic, neuropathy, leprosy, charcot neuroarthropathy

## Abstract

Leprosy is a chronic infection of the skin, eyes, and peripheral nerves due to the slow-growing, acid-fast bacillus *Mycobacterium leprae*. Devastating complications include Charcot neuroarthropathy and insensate hands and feet. We present the case of an 81-year-old female with rheumatoid arthritis and 50 years of polar lepromatous leprosy who suffered from bilateral collapsed arches, flat feet, and bone deformities of Charcot feet.

## Introduction

Here, we present a patient with underlying rheumatoid arthritis and polar lepromatous lepromatous leprosy who suffered from Charcot neuroarthropathy with insensate feet and outline the developmental connection between the two conditions.

## Case presentation

**Figure 1:**
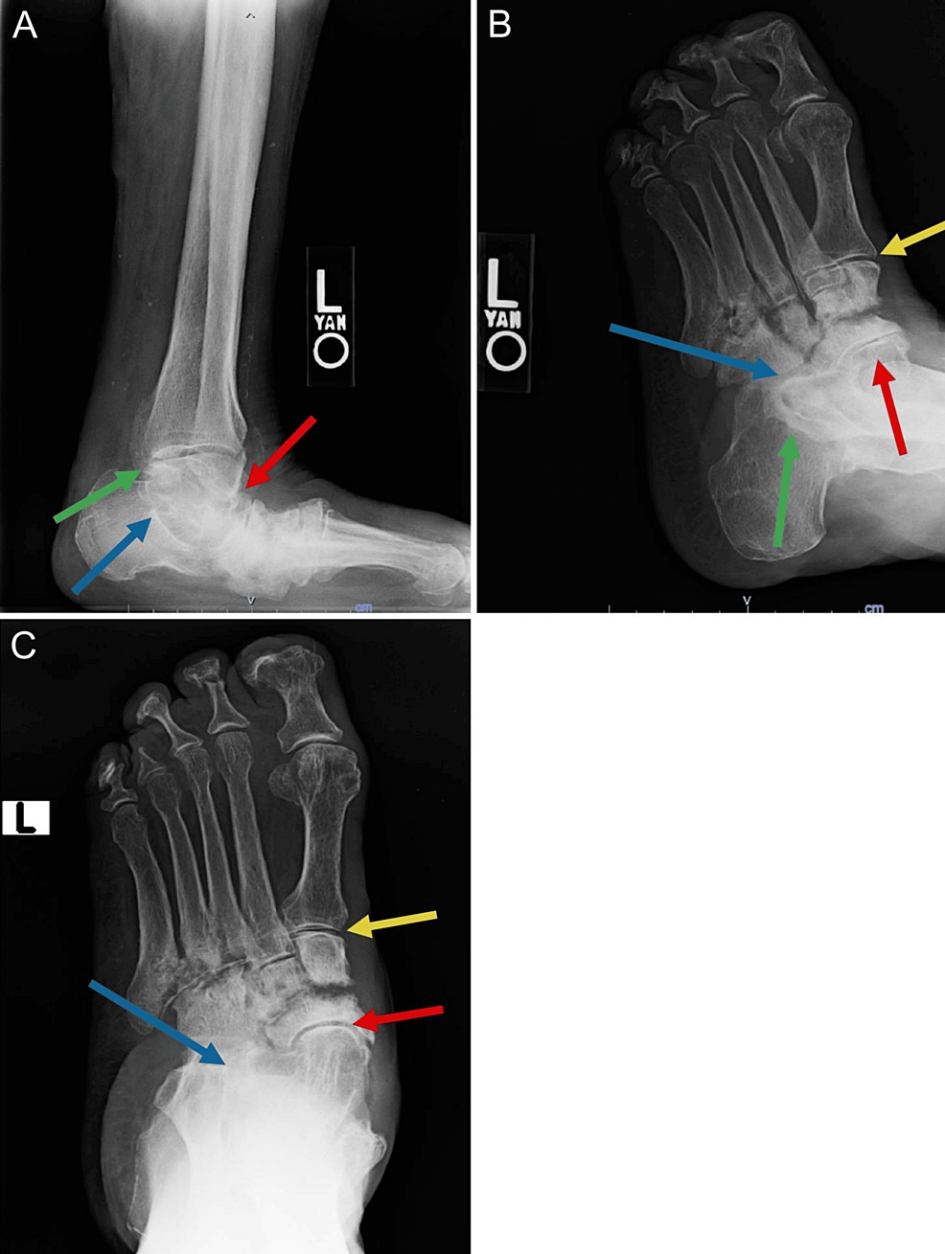
X-ray of the patient's left foot showing the (A) lateral view, (B) lateral superior view, and (C) superior view of Charcot neuroarthropathic osteolysis of the talonavicular (red arrow), talocalcaneal (green arrow), and calcaneocuboid (blue arrow) joints as well as the Lisfranc joint (yellow arrow)

**Figure 2:**
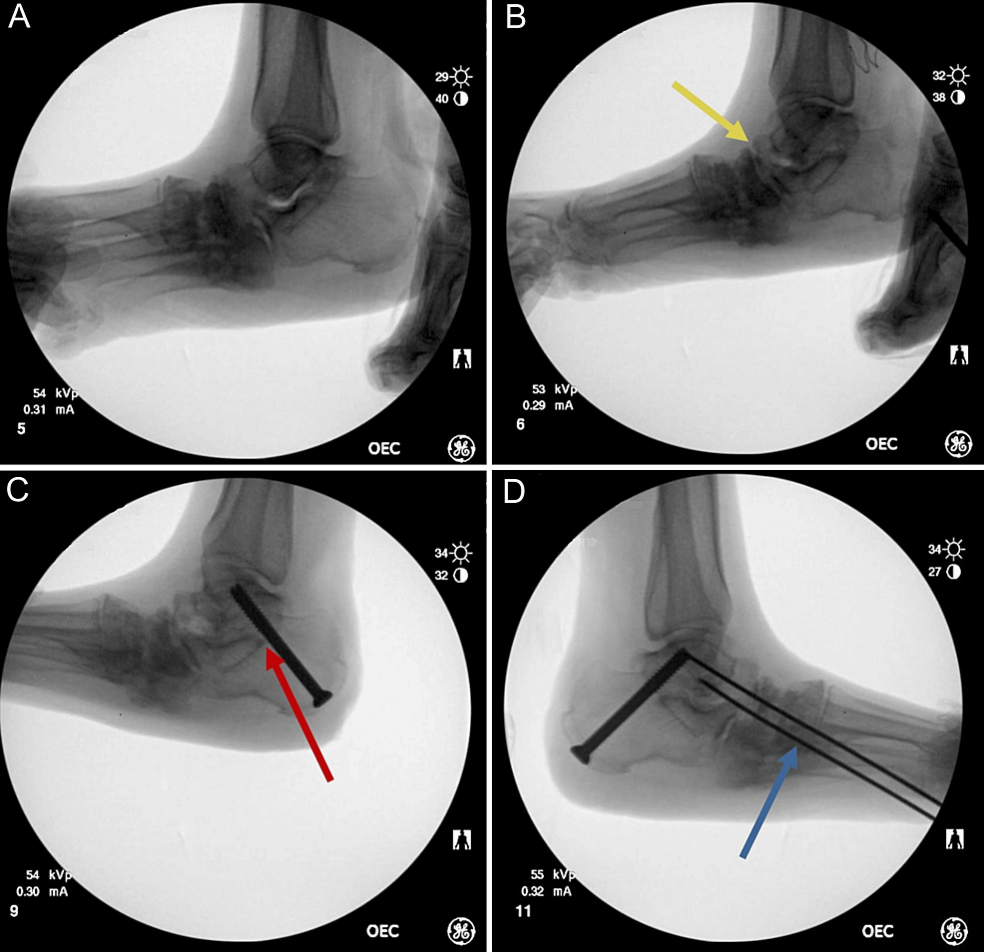
Fluoroscopic image of the patient's left foot showing the (A) lateral superior view, (B) lateral view of the Charcot neuroarthropathic osteolysis (yellow arrow), (C) subtalar arthrodesis (red arrow), and (D) pin fixation of distal degraded joints correcting the rigid pes planovalgus deformity (blue arrow)

An 81-year-old female with a medical history including Hansen's disease, hypothyroidism, and rheumatoid arthritis presented for scheduled left foot irrigation and debridement, midfoot and subtalar joint arthrodesis, and medical column osteotomies with application of an external fixator. She was first diagnosed with subtype polar lepromatous leprosy at age 30, and after a treatment regimen including dapsone (16 years), rifampin (seven years), minocycline (12 years), and clarithromycin (14 years), the patient was considered cleared at age 70. Her leprosy sequelae include severe sensory impairment in all four limbs, causing prior bilateral foot deformity and wound formation, as well as Charcot neuroarthropathy and rigid pes planovalgus in the left lower extremity, as shown in Figure 1A-1C. Previously, conservative treatments such as offloading and multiple irrigation and debridements with local exostectomies failed to sustain long-term remission of the left plantar medial midfoot or create a stable plantigrade foot. Most recently, the patient underwent an exostectomy 10 weeks prior, following another re-ulceration of the left plantar wound site. After exhausting other treatments (Figure 2A, 2B), the patient opted for flatfoot reconstruction to address the gross deformities contributing to her chronic ulcer and limited mobility, which consisted of a subtalar arthrodesis to support the talocalcaneal joint (Figure 2C) as well as two separate K-wire fixations of the talonavicular and calcaneocuboid joints, each through the Lisfranc joint to support these degraded joints (Figure 2D).

Following the procedure, the patient was admitted to the hospital for postoperative pin external fixator management. After an unremarkable hospital course, she was discharged on postoperative day 2. At the one-week follow-up, the patient presented with clean pin sites, an intact surgical incision, an ulceration measuring 1.0 cm × 1.0 cm × 0.2 cm, and foot in rectus. At the three-week follow-up, the only changes were a smaller wound measuring 0.5 cm × 0.5 cm × 0.2 cm.

## Discussion

The manifestations of leprosy are caused by an infection with *Mycobacterium leprae* (*M. leprae*), which predominantly (in 95% of recent novel cases) occurs in just 14 countries, more than 60% of which are in India [1]. It is possible to cure with early detection and treatment using multiple-drug therapy; however, treatment becomes increasingly difficult with later detection and often leads to numerous and multifaceted consequences [1]. The bacteria is known to primarily infect macrophages and Schwann cells, the latter of which commonly results in neuritis as well as motor, autonomic, and sensory peripheral neuropathy [2]. Bone lesions are also commonly noted in patients with leprosy, most notably the destruction of phalangeal joints in the hands and feet thought to be related to the direct effects of the bacteria [3].

Charcot neuroarthropathy affects up to 35% of individuals with peripheral neuropathy and is characterized by the osteolytic destruction of pedal bones and joints, which significantly alters the structure of the foot [4]. The exact pathophysiology of Charcot neuroarthropathy is not fully understood; however, there are a number of accepted theories that are not mutually exclusive [4].

The neuro-bone-inflammatory theory posits that the repetitive trauma that arises from the natural manifestation of peripheral neuropathy induces an inflammatory response involving cytokines such as interleukin-6 and tumor necrosis factor-α that upregulate osteolytic activity via numerous pathways [4]. The neuro-traumatic theory asserts that the repetitive microtraumas that result from motor neuropathy-induced atypical biomechanics lead to osteo-destruction. Finally, the neurovascular theory contends that increased blood flow due to damaged trophic nerves results in bone degradation [4].

The causal chain of events from the initial bacterial infection to the development of chronic Charcot neuropathy can be inferred from the established consequences of leprosy and the theories describing the cause(s) of Charcot neuroarthropathy. However, it is important to acknowledge the factors that likely contributed to the development of the chronic progressive infection of *M. leprae* such as inadequate therapy in response to the acute infection and possible antibiotic resistance.

The binding of *M. leprae* to Schwann cells leads to a reduction of myelination and associated neuropathic effects [5]. Autonomic, motor, and sensory neuropathies produce compounding symptomatology that exacerbates a number of secondary conditions. The lack of fine motor control in the lower extremities presents as atypical pedal biomechanics, which produce repeated shear and normal force microtraumas. Without sensory feedback, the biomechanical adjustments that mitigate these microtraumas are minimized and the reduction in sudoriferous activity exacerbates their effects [6]. After extensive, repeated microtraumas, many patients develop foot ulcerations. Moreover, a subset of the patient population develops Charcot neuroarthropathy; it is proposed that the induced inflammatory response from the microtraumas upregulates osteoclast precursor differentiation and osteolytic activity [7-10]. This osteolysis and inflammation sometimes result in a characteristic plantar shift of the mid-pedal bones, often referred to as "rocker-bottom foot," as well as other physical distortions [11]. This effect may be further exacerbated by the potential osteolytic action of the *M. leprae *bacteria, which is hypothesized to result from the downregulation of the phosphate-regulating gene with homologies to endopeptidase on the X chromosome (*PHEX*), a gene with modulatory effects on bone matrix mineralization, phosphate renal excretion, serum levels of fibroblast growth factor 23 (FGF23) and 1,25(OH)2 vitamin D regulation [12].

## Conclusions

This progression makes it clear that patients with leprosy are at an elevated risk of developing Charcot neuroarthropathy. In cases where eradication of the bacterial infection is not possible, therapies to reduce the risk of secondary Charcot neuroarthropathy development should be employed, such as offloading and using anti-inflammatory treatment to mitigate peripheral trauma and inflammation. Here, we show that monitoring patients with leprosy for distal joint degradation will lead to early detection, surgical correction, and offloading, which increases the likelihood that the patient will not require extensive reconstruction or amputation.
